# Antibacterial Activity and Action Mechanism of the Essential Oil from *Enteromorpha linza* L. against Foodborne Pathogenic Bacteria

**DOI:** 10.3390/molecules21030388

**Published:** 2016-03-21

**Authors:** Jayanta Kumar Patra, Kwang-Hyun Baek

**Affiliations:** 1 Research Institute of Biotechnology & Medical Converged Science, Dongguk University, Ilsandong-gu, Gyeonggi-do 10326, Korea; jkpatra.cet@gmail.com; 2 School of Biotechnology, Yeungnam University, Gyeongsan, Gyeongbuk 712-749, Korea

**Keywords:** antibacterial property, *Bacillus cereus*, *Enteromorpha linza*, essential oil, seaweed, *Staphylococcus aureus*

## Abstract

Foodborne illness and disease caused by foodborne pathogenic bacteria is continuing to increase day by day and it has become an important topic of concern among various food industries. Many types of synthetic antibacterial agents have been used in food processing and food preservation; however, they are not safe and have resulted in various health-related issues. Therefore, in the present study, essential oil from an edible seaweed, *Enteromorpha linza* (AEO), was evaluated for its antibacterial activity against foodborne pathogens, along with the mechanism of its antibacterial action. AEO at 25 mg/disc was highly active against *Bacillus cereus* (12.3–12.7 mm inhibition zone) and *Staphylococcus aureus* (12.7–13.3 mm inhibition zone). The minimum inhibitory concentration and minimum bactericidal concentration values of AEO ranged from 12.5–25 mg/mL. Further investigation of the mechanism of action of AEO revealed its strong impairing effect on the viability of bacterial cells and membrane permeability, as indicated by a significant increase in leakage of 260 nm absorbing materials and K^+^ ions from the cell membrane and loss of high salt tolerance. Taken together, these data suggest that AEO has the potential for use as an effective antibacterial agent that functions by impairing cell membrane permeability via morphological alternations, resulting in cellular lysis and cell death.

## 1. Introduction

Reports on foodborne illness and disease caused by consumption of food contaminated by foodborne pathogens are increasing day by day throughout the world [1,2]. Food spoilage, food poisoning and other food-related diseases have become an important topic of concern among various food industries [3,4]. Different synthetic additives and antimicrobial agents are continuously used during the processing of food to avoid contamination and increase of shelf-life by diminishing the growth of microorganisms [5]. However, there is increasing consumer concern regarding the safety of the synthetic chemicals used to preserve foods and their side effects. Much attention is being given throughout the world to minimize the use of synthetic antibacterial agents in food processing and food preservation. Therefore, there has been increasing interest in identifying natural and safe antibacterial compounds from various natural sources.

Plant-based essential oils have been widely applied for a variety of purposes for thousands of years [6,7]. Essential oils or volatile oils extracted from plants are aromatic oily liquids composed of a mixture of phenolic compounds, terpenoids, alcohols, aldehydes and other important bioactive compounds [8]. Essential oils often have antibacterial, antifungal, insecticidal, antioxidant and anti-inflammatory properties [5,9,10,11] and have therefore been used in the preservation of post-harvest crops and food [12]. Owing to the antimicrobial properties of essential oils from various plants, they have potential applications in the preservation of raw and processed food, as well as in pharmaceutical, natural and alternative medicine [7]. 

Currently, many essential oils from various plants and their parts have been used in the pharmaceutical, perfume, cosmetic and food industries [13,14,15]. However, there are few reports on essential oils from marine algae and their potential antimicrobial applications, despite the ease and large-scale cultivation of marine algae. Nevertheless, marine algae have been shown to have pharmaceutical properties; therefore, there is growing interest regarding the use of the active compounds from marine algae in various applications of prospective pharmaceuticals. *Enteromorpha linza* (L.) J. Ag. is an edible, green, broad paddle–shaped seaweed commonly seen in Asian, North European and Mediterranean coastal areas [16]. The seaweed is rich in various bioactive compounds, and few of its medicinal potentials have been reported [17,18]. Therefore, in this study, we evaluated the antibacterial potential of essential oil from *Enteromorpha linza* L. (AEO) against foodborne pathogens, as well as its mechanism of action.

## 2. Results

### 2.1. Antibacterial Activity of AEO

The antibacterial potential of AEO against two different foodborne bacterial pathogens is presented in Table 1. The results revealed that AEO exerted moderate bactericidal activity against *S. aureus* and *B. cereus* with zones of inhibition ranging between 12.3 and 13.3 mm (Table 1). Rifampicin, a standard antibiotic, showed higher bactericidal activity against all four strains, whereas the negative control dimethyl sulfoxide (DMSO) showed no inhibitory activity. The minimum inhibitory concentration (MIC) and minimum bactericidal concentration (MBC) value of AEO against all tested bacteria ranged from 12.5 to 25 mg/mL (Table 2).

### 2.2. Viability of Bacterial Cells

**Figure 1:**
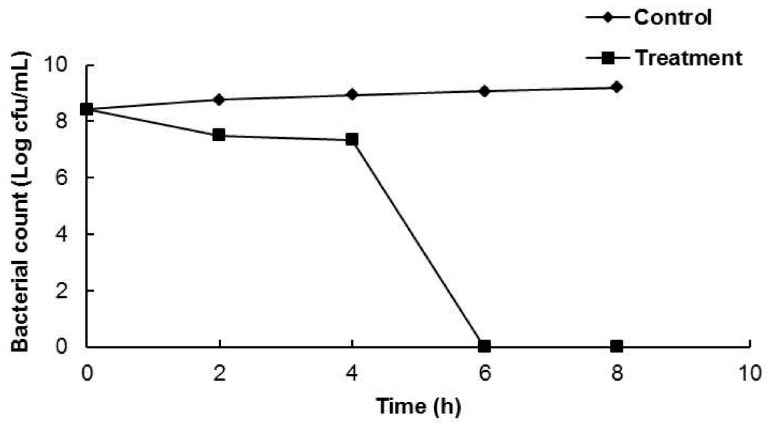
Effect of *Enteromorpha linza* essential oil (AEO) at MIC concentration on the viability of *Bacillus cereus* ATCC 13061. Data are expressed as the mean ± SD.

One foodborne bacterial strain (*B. cereus* ATCC 13061) was selected for a further in-depth study of the antibacterial mode of action of AEO against the tested foodborne pathogens. The effects of AEO on cell viability are presented in Figure 1. AEO at the MIC concentration did not show any significant reduction in viable cell count of the bacteria until 4 h of incubation; however, it controlled the growth of viable cells completely after 6 h of incubation (Figure 1).

### 2.3. Effect on Release of 260 nm Absorbing Materials

**Figure 2:**
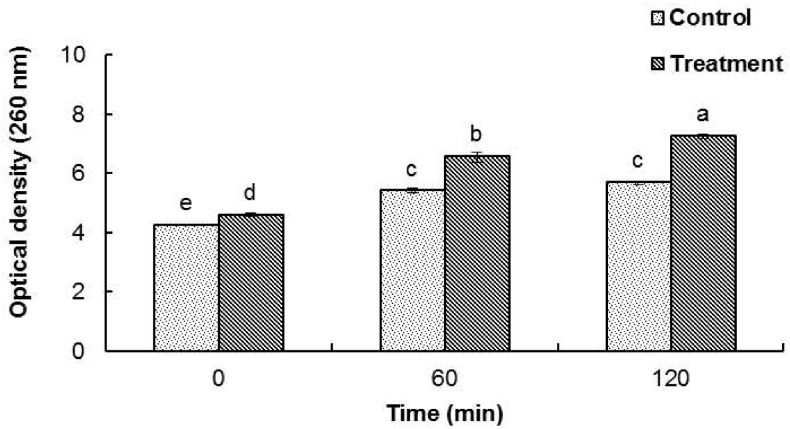
Effect of *Enteromorpha linza* essential oil (AEO) at MIC concentration on the release of 260 nm absorbing material of *Bacillus cereus* ATCC 13061. Data are expressed as the mean ± SD. Values with different superscript letters are significantly different at *p* < 0.05.

The release of cellular materials such as nucleic acids, proteins and metabolites from *B. cereus* ATCC 13061 treated with AEO is presented in Figure 2. When *B. cereus* ATCC 13061 was treated with the MIC concentration of AEO, there was a continual increase in the concentration of 260 nm absorbing materials over 120 min of incubation (Figure 2). The control treated with DMSO did not show an increase in the concentration of 260 nm absorbing materials after 60 min of incubation.

### 2.4. Permeability of Cell Membrane and Leakage of Potassium Ion

**Figure 3:**
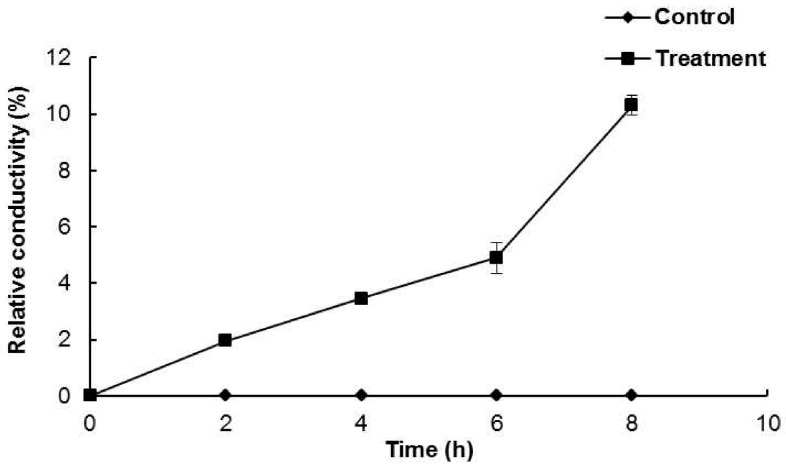
Effect of *Enteromorpha linza* essential oil (AEO) at MIC concentration on permeability of the cell membrane of *Bacillus cereus* ATCC 13061. Data are expressed as the mean ± SD.

The permeability of the cell membrane was measured based on the electrical conductivity and leakage of potassium ions from *B. cereus* ATCC 13061 treated with DMSO or AEO. The control treated with DMSO showed no change in relative conductivity over 8 h of incubation; however, *B. cereus* ATCC 13061 treated with AEO at the MIC concentration showed a steady increase in electrical conductivity (Figure 3). Furthermore, after 6 h of incubation, there was a sharp increase in electrical conductivity (Figure 3), which corresponded to the complete loss of viability of *B. cereus* ATCC 13061 treated with the MIC concentration of AEO after 6 h of incubation as shown in Figure 1.

**Figure 4:**
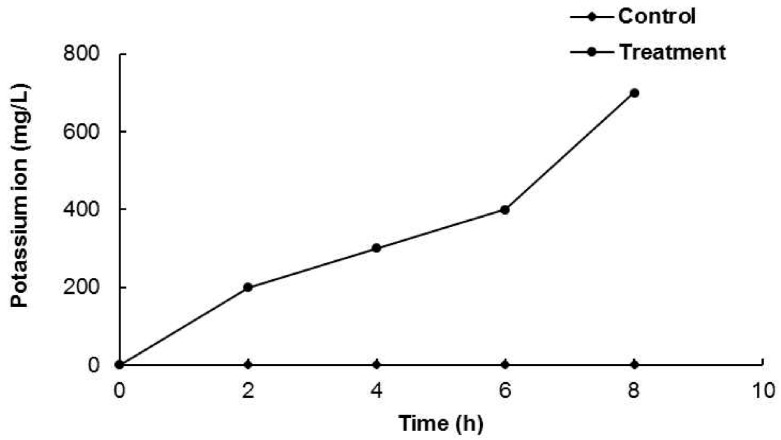
Effect of *Enteromorpha linza* essential oil (AEO) at MIC concentration on leakage of potassium ions from *Bacillus cereus* ATCC 13061.

Another parameter for permeability impairedness can be the leakage of K^+^ ions from cells. When *B. cereus* ATCC 13061 was treated with AEO at the MIC concentration, the leakage of potassium ions was observed (Figure 4). As shown in Figure 4, *B. cereus* ATCC 13061 treated with AEO showed continual leakage of K^+^ ions, especially with a sharp increase after 6 h of incubation (Figure 4), which also corresponded with a sharp increase in the cell membrane permeability after 6 h of incubation as shown in Figure 3.

### 2.5. Loss of Salt Tolerance Capacity

**Figure 5:**
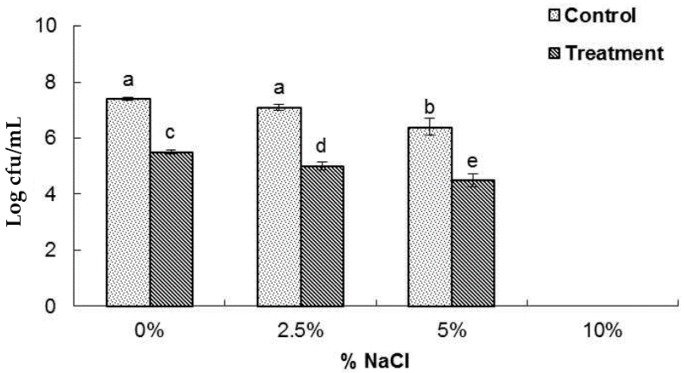
Effect of *Enteromorpha linza* essential oil (AEO) at MIC concentration on the reduction of salt tolerance of *Bacillus cereus* ATCC 13061. Data are expressed as the mean ± SD. Values with different superscript letters are significantly different at *p* < 0.05.

The salt tolerance potential of *B. cereus* ATCC 13061 treated with AEO at the MIC concentration is shown in Figure 5. When the bacteria pretreated with AEO or DMSO were inoculated on nutrient agar media supplemented with different concentrations of NaCl (0%, 2.5%, 5% and 10%), a significant decrease in the number of colony-forming units at each concentration was observed in the AEO-pretreated bacteria relative to those pretreated with DMSO (Figure 5). Neither the control nor the treated sample showed any growth of bacteria in the NA plates supplemented with 10% NaCl.

### 2.6. SEM Analysis

**Figure 6:**
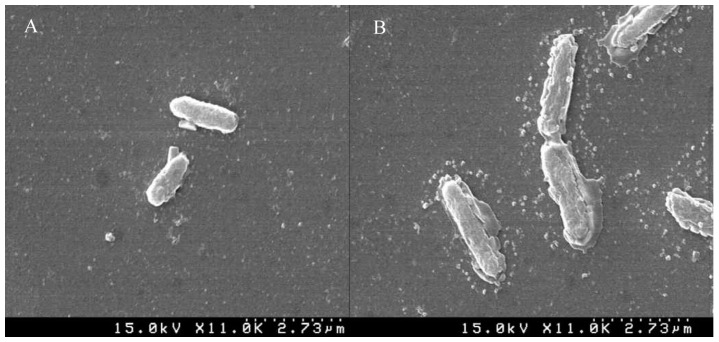
Scanning electron microscopy of *Bacillus cereus* ATCC 13061. (**A**): untreated *B. cereus*; (**B**): *B. cereus* treated with the essential oil from *Enteromorpha linza*.

The effect of AEO on the cellular structure of the bacteria was further visualized by SEM analysis (Figure 6). The cells of *B. cereus* ATCC 13061 treated with AEO at the MIC concentration showed abnormal morphology accompanied with longer and swollen shapes relative to the control with a regular and smooth surface (Figure 6).

## 3. Discussion

Decrease in the nutritional quality of food, food poisoning, food spoilage and other food-related diseases are primarily caused by a group of foodborne pathogens [9,11,20]. Synthetic chemicals and physical methods are being used by various food industries to prevent contamination and preserve food from these harmful pathogens [21]. However, many of the techniques and chemicals applied are found to be ineffective or result in different health issues and side effects, eventually affecting the safety of food as well as that of the consumers. Increased awareness among the consumers regarding the use of natural products in food preservation has forced the food industries to search for alternative natural preservatives with potential for use in food processing and preservation while increasing the shelf-life of food items. 

Seaweeds are rich in protein, vitamins and minerals and are extensively consumed by people of many countries, especially in South Korea, Japan and China, in both fresh and dry form [22]. Many seaweeds have been found to have antioxidant, antimicrobial, anticoagulant, and anti-ulcer activities [23,24]. However, extraction of essential oils from these edible seaweeds and their potential bactericidal activity against various foodborne pathogens has not been intensively investigated. Therefore, through this research, the antibacterial potentials of essential oil from the fresh edible seaweed *E. linza* L. against two dominant foodborne bacteria and their modes of action were investigated.

AEO displayed potent antibacterial activity against both *B. cereus* (ATCC 10876 and ATCC 13061) and *S. aureus* (ATCC 12600), *S. aureus* ATCC 49444 (reclassified as *S. pseudintermedius*) [19] (Table 1). Previously, the antimicrobial potentials of different extracts and essential oil from various seaweeds against different types of microorganisms were reported [17,19,22,25,26], and our research also verified the effectiveness of the essential oil from *E. linza* on controlling two devastating foodborne pathogens. The antibacterial potential of AEO against *B. cereus* and *S. aureus* might be due to the presence of a number of active compounds such as hexadecanoic acid, nonadecadiene, tetradecanoic acid, tridecanol and azetidine, *etc.*, in the AEO, as evident from the GC-MS analysis [27]. A possible mechanism for the antibacterial effects of AEO might be the hydrophobic properties of essential oils that easily penetrate into the bacterial cells due to the structure of the cell wall of Gram-positive bacteria eventually causing cellular death [28].

The mode of action of AEO against the investigated foodborne bacteria was revealed by several assays, including cell viability, leakage of 260 nm absorbing materials, cell membrane leakage, loss of salt tolerance and direct observation of cell morphology by SEM using *B. cereus* ATCC 13061 as the model system. The bacterial cells treated with AEO at the MIC concentration did not have any viable cells after 6 h of co-incubation (Figure 1), which might have been due to the easy penetration of AEO into the bacterial cell resulting in complete lysis and cellular death [28].

A significant increase in the optical density at 260 nm was observed in the bacterial solution treated with AEO (Figure 2), indicating a more active release of intercellular materials such as nucleic acids to the outer solution by cellular leakage. A previous study revealed that essential oils caused hole-like deformities in the cell surface, resulting in cellular leakage [9,29]. The bacterial cytoplasmic membrane provides a selective barrier to small ions such as K^+^, Ca^2+^, Na^+^, preventing entry and exit from the cells. This control of permeability by the cellular membrane is the key regulatory factor for various cellular functions, including cell metabolism maintenance, solute transport, energy transduction processes, *etc.* [30,31]. The permeability of the cytoplasmic membrane of *B. cereus* ATCC 13061 was significantly impaired by the addition of AEO into the culture of *B. cereus* as indicated by the significant increase in the relative electrical conductivity and the concentration of K^+^ ions in the bacterial solution (Figure 3 and Figure 4). Hydrophobicity is an important property of essential oils that leads to the accumulation of oils inside bacterial cell membranes, resulting in cellular lysis by disturbance of their structure and increased cellular leakage [32,33,34]. The loss of the osmoregulation potential of bacterial cells to salts by the addition of AEO was observed (Figure 5). This loss of salt tolerance is additional strong evidence confirming damage to the membrane of bacterial cells due to the accumulation of essential oil in them [35]. 

*B. cereus* ATCC 13061 treated with AEO at the concentration of MIC experienced severe morphological changes (Figure 6), which again confirmed the mechanism of antibacterial action of AEO. Clear morphological alterations in the cell wall of bacteria treated with essential oils have been reported in many studies [36,37,38,39,40]. These changes in the physical and morphological characteristics of the bacterial cells explain the effect of AEO on their integrity and the permeability of the bacterial cell eventually causing cellular lysis and death.

## 4. Materials and Methods 

### 4.1. Chemicals and Instruments

All chemicals, including rifampicin, were purchased from Merck (Rahway, NJ, USA) or Sigma-Aldrich (St. Louis, MO, USA). A spectrophotometer (ASP 3700, ACTGene Inc., Piscataway, NJ, USA), conductivity meter (Con 6, LaMotte, Chestertown, MD, USA) and scanning electron microscope (S-4110, Hitachi, Tokyo, Japan) were used for spectrophotometric analysis, conductivity measurement and microscopic observation, respectively.

### 4.2. Extraction of Essential Oil from E. linza

Fresh green seaweed of *E. linza* was purchased from a local market in Gyeongsan, Gyeongbuk, Korea. A total of 500 g of seaweed was washed with distilled water and then put in a 10 L glass container. Next, 5 L of distilled water was added and the sample was subjected to hydro-distillation using a micro-wave assisted extraction machine manufactured by KMD Engineering (Paju, Korea) for 4 h. The operation conditions were an oven power capacity of 40 W and frequency of 15 gkH. The collected distillate was mixed with dichloromethane in a separating funnel, shaken vigorously and kept until the samples settled. The lower layer of the separating funnel was collected and concentrated using a rotary evaporator (N-1110, EYELA, Tokyo Rikakikai Co., Ltd., Tokyo, Japan) at 40 °C. The algae essential oil (AEO) was then dried over anhydrous sodium sulfate and kept in a tightly closed vial at 4 °C until use. The AEO was obtained as a light yellow transparent liquid with a 0.32% yield.

### 4.3. Foodborne Bacterial Pathogens

Four different foodborne bacterial pathogens from two species were used in the present study. These strains included *Bacillus cereus* (ATCC 10876 and ATCC 13061) and *Staphylococcus aureus* (ATCC 12600 and ATCC 49444). The bacterial pathogens were grown on nutrient broth media (Difco; Becton, Dickinson and Company, Sparks Glencoe, MD, USA).

### 4.4. Determination of Antibacterial Potential of AEO

Prior to use, AEO was diluted to 50% (*v*/*v*) in 5% dimethylsulphoxide (DMSO) and then sterilized through a 0.22 µm nylon syringe filter. The antibacterial potential of AEO was evaluated against the four foodborne pathogens using the standard disc diffusion method [1]. A total 100 µL of suspension containing 10^7^ cfu/mL of the four foodborne bacterial pathogens was uniformly spread on separate nutrient agar (NA) plates. Paper discs (6 mm, Advantec, Toyo Roshi Kaisha Ltd., Tokyo, Japan) with 25 mg/disc of AEO were placed on the inoculated agar. Rifampicin (20 µg/disc) was used as a positive control and 5% DMSO as a negative control. The diameters of zones of inhibition were measured after 24 h of incubation at 37 °C.

The minimum inhibitory concentration (MIC) and minimum bactericidal concentration (MBC) were determined by the two-fold dilution method, with minor modifications as described by Kubo *et al.* [41]. The lowest concentration of AEO not showing any visible growth of test organisms was determined as the MIC value and the concentration showing complete absence of growth of bacterial colonies on the agar surface was defined as the MBC value (mg/mL).

### 4.5. Viability of Bacterial Cell

The antibacterial effect of AEO on the viability of the bacterial pathogens with respect to incubation time was evaluated by the time kill assay [42]. Only one foodborne bacterium, *B. cereus* (ATCC 13061), was selected for the assay. Bacterial culture with AEO at MIC concentration and bacterial cultures with 5% DMSO were used as the treatment and control, respectively. One mL of the bacteria culture (10^7^ cfu/mL) was added to the assay and total volume was 10 mL. The cultures were incubated at 37 °C for 8 h, during which time bacterial samples were taken from the incubator at every 2 h, serially diluted in phosphate buffer saline, spread on the surface of nutrient agar plates and incubated at 37 °C for 24 h. Subsequently, the colonies were counted in terms of colony-forming units (cfu) per mL of sample.

### 4.6. Integrity of the Cell Membrane

The integrity of the cell membrane of *B. cereus* (ATCC 13061) was determined by the degree of cellular materials, especially DNA and RNA, in the supernatant [32]. AEO at the MIC concentration was added to 2 mL of the bacterial inoculum (10^7^ cfu/mL) in sterilized peptone water (0.1 g/100 mL) and then incubated at 37 °C. Bacterial samples were collected after 0, 60 and 120 min of incubation and centrifuged at 1372 *g*, after which the UV absorbance of the supernatant was measured at 260 nm using a spectrophotometer. Corrections for treated cultures were made by taking AEO without bacteria in sterilized peptone water. The untreated bacterial cultures were corrected with sterile peptone water.

### 4.7. Permeability of the Cell Membrane

The effect of AEO on bacterial cell membrane permeability in terms of relative electrical conductivity was determined according to the standard method described by Kong *et al.* [43]. Prior to the experiment, *B. cereus* (ATCC 13061) was subcultured in nutrient broth media and incubated at 37 °C for 10 h. Following incubation, the bacterial pathogen were centrifuged at 2800 *g* for 10 min, and then washed with 5% glucose until its electrical conductivity was near to that of 5% glucose as measured in a conductivity meter (Con 6; LaMotte Company, Chestertown, MD, USA). AEO at the MIC concentration was added to 5% glucose and its electrical conductivity was determined (L_1_). MIC concentration of AEO was added to the isotonic bacterial solution, mixed completely and incubated at 37 °C for 8 h, during which time the electrical conductivity of the AEO-bacterial mixture was measured every 2 h (L_2_). The control was the killed bacteria in 5% glucose by being treated in boiling water for 5 min. The electrical conductivity of the control was marked as L_0_. The permeability of the bacteria membrane in terms of its relative electrical conductivity was calculated as follows:

Relative conductivity (%) = 100 × [(L_2_ − L_1_)/L_0_] (1)

### 4.8. Leakage of Potassium Ion

The leakage of potassium (K^+^) ion into the bacterial suspension was determined as described by Paul *et al.* [44]. The leakage of free K^+^ ion in the bacterial suspension of *B. cereus* (ATCC 13061) was detected after exposing the bacteria (10^7^ cfu/mL) to the MIC concentration of AEO in sterile peptone water (0.1 g/100 mL) and incubating the samples at 37 °C for 8 h, during which time the extracellular potassium concentration was measured every 2 h using a Kalium/Potassium kit (Quantofix, Macherey-Nagel GmbH & Co. KG, Duren, Germany). A culture flask without AEO was used as a control. All data were reported as the amount of free K^+^ ion (mg/L) in the bacterial suspension at each time interval.

### 4.9. Loss of Salt Tolerance

The salt tolerance effect of *B. cereus* (ATCC 13061) treated with the MIC concentration of AEO was evaluated on nutrient agar plates supplemented with different concentrations of NaCl [35]. Prior to use, *B. cereus* was cultured fresh and incubated at 37 °C for overnight. After incubation, the overnight culture of *B. cereus* were treated with AEO and further incubated for 60 min at 37 °C. Then the samples were serially diluted and inoculated on NA plates supplemented with different concentrations of NaCl (0%, 2.5%, 5.0% and 10.0%). Bacterial culture without AEO was used as the control for each NA-NaCl plate. After incubation, both the control and treatment plates were compared, and the results were expressed in terms of Log10 cfu/mL. The experiment was repeated three times.

### 4.10. Scanning Electron Microscopy (SEM) Analysis

The effects of the essential oil on the morphology of *B. cereus* (ATCC 13061) were determined by SEM [30]. Overnight cultures of both control and treated samples were centrifuged at 1000× *g* for 10 min, after which the pellet was washed slowly with 50 mM phosphate buffer solution (pH 7.2), mounted over glass slides and fixed with 100 mL glutaraldehyde (2.5%). The specimen was then dehydrated using different concentrations of ethanol (50%–100%). Finally, ethanol was replaced by *t*-butanol and incubated at room temperature for 2 h. Finally, the specimen was sputter-coated with platinum in an ion coater for 120 s and observed by SEM (S-4100; Hitachi).

### 4.11. Statistical Analysis

The results were expressed as the mean ± standard deviation (SD) and the statistical analysis were done using Statistical Analysis Software (SAS) version 9.4 (SAS Inc., Cary, NC, USA). One way ANOVA and Duncan’s multiple range tests were done and the results were considered significant at probability *p < 0.05*.

## 5. Conclusions

The results of the present study indicate that AEO from the edible seaweed *E. linza* can be a potential alternative antibacterial agent that can be used as an additive in food to act against foodborne pathogens. Study on the mechanism of action of AEO indicated that it influences the permeability of the cellular membrane by binding to the cell surface and then penetrating the bacterial cell membrane, thereby resulting in cellular lysis and cell death.

## Figures and Tables

**Table 1:** Evaluation of antibacterial activity of *Enteromorpha linza* essential oil against selected foodborne bacteria.

Bacterial Pathogens	Diameter of Inhibition Zone (mm)	
	Essential Oil **	Standard ***
*Bacillus cereus* ATCC 10876	12.7 ± 1.5 ^a,^*	24.0 ± 2.8 ^b^
*B. cereus* ATCC 13061	12.3 ± 0.6 ^a^	27.7 ± 0.6 ^a^
*Staphylococcus aureus* ATCC 49444 (reclassified as *S. pseudintermedius*) [19]	13.3 ± 1.5 ^a^	24.5 ± 0.7 ^b^
*S. aureus* ATCC 12600	12.7 ± 0.6 ^a^	27.7 ± 0.6 ^a^

* Data are expressed as the means ± standard deviation. Values in the same column with different superscripts are significantly different at *p* < 0.05; ** Essential oil at 25 mg/disc; *** Standard was rifampicin at 20 µg/disc*.*

**Table 2:** Determination of MIC and MBC values of *Enteromorpha linza* essential oil against selected foodborne bacteria.

Bacterial Pathogens	MIC *	MBC *
*B. cereus* ATCC 10876	25	25
*B. cereus* ATCC 13061	12.5	12.5
*S. aureus* ATCC 49444 (reclassified as *S. pseudintermedius*) [19]	12.5	12.5
*S. aureus* ATCC 12600	12.5	25

* Data are expressed in mg/mL.
